# Comment on “Effects of Yoga on Utero-Fetal-Placental Circulation in High-Risk Pregnancy: A Randomized Controlled Trial”

**DOI:** 10.1155/2015/587489

**Published:** 2015-09-15

**Authors:** Siddharudha Shivalli

**Affiliations:** Department of Community Medicine, Yenepoya Medical College, Yenepoya University, Mangalore, Karnataka 575018, India

I read the paper titled “Effects of Yoga on Utero-Fetal-Placental Circulation in High-Risk Pregnancy: A Randomized Controlled Trial” by A. Rakhshani et al. [1] with a great interest. Authors' efforts are highly commendable. The strength of this study, apart from those mentioned by the authors, is the objectiveness of the key outcomes for yoga intervention. However, the following issues and concerns need to be addressed.

Authors mention that the “sample profile matched closely that of the Bengaluru metropolitan population” which is considered as one of the strengths of the study. I feel that it has to be justified with proper reference/s as the analyzed sample size was too small (*n* = 53, Table 2). In fact, the issue of smaller sample size has been mentioned in the limitation.

Authors have used outcome measures such as fetal biparietal diameter, head circumference, abdominal circumference, femur length, and estimated fetal weight (Table 3). However, maternal hemoglobin, anemia status, physical activity, dietary, and iron-folate/other supplement intake have an effect on all these fetal parameters [2]. These are the potential confounders in this study and they must have been addressed.

Nonetheless, this study highlights the need of higher level of evidence to assess the effect of yoga on maternal and fetal outcomes.
